# Mutual Influence of Parental Depression and Parenting: An Actor–Partner Interdependence Analysis Based on Chinese Families with Adolescent Twins

**DOI:** 10.3390/bs16010103

**Published:** 2026-01-12

**Authors:** Min Zhou, Bingtian Li, Xinying Li, Jie Chen

**Affiliations:** 1 State Key Laboratory of Cognitive Science and Mental Health, Institute of Psychology, Chinese Academy of Sciences, Beijing 100101, China; zhoum@psych.ac.cn (M.Z.); libt@psych.ac.cn (B.L.); lixy@psych.ac.cn (X.L.); 2 Department of Psychology, University of Chinese Academy of Sciences, Beijing 100049, China; 3 The Affiliated High School of Peking University Fengtai Campus, Beijing 100078, China

**Keywords:** depression, parenting, actor–partner interdependence model, adolescent

## Abstract

Examining the dyadic effects of parental depression on parenting behaviors is important for understanding the dynamic impact of a family member’s negative emotions on parenting and family-based interventions. To clarify the interpersonal processes between parental depression and parenting within families, this study aimed to investigate the mutual influence of parental depression and parenting (warmth–reasoning and harshness–hostility) in one large sample of adolescent twins and their parents. A sample of 1387 Chinese families with adolescent twins was used. The actor–partner interdependence model (APIM) was used to examine the mutual influences. By examining the dyadic patterns with APIM, we found that depressive symptoms in mothers or fathers significantly influence their own and their partner’s parenting behaviors. The model comparisons found no significant difference in the partner effect between maternal and paternal depression. In the Chinese family system, depression in one parent influences not only their own parenting but also their partner’s parenting.

## 1. Introduction

The Family System Theory (FST) posits that families are complex, integrated ecosystems comprising multiple subsystems. These include marital subsystems (husband and wife), sibling subsystems, and parent–child subsystems. Individuals and subsystems can exert reciprocal influences in complex ways (Cox & Paley, 2003). Therefore, there is a possibility that depression in one parent affects not only their own parenting, but also their partner’s parenting. This mutual influence of a couple’s depression and parenting can be examined by the Actor–Partner Interdependence Model (APIM) (Kenny et al., 2006). In this model, the actor effect refers to the relation of a parent’s depression to their own parenting, and the partner effect refers to the influence of a parent’s depression on a partner’s parenting. Only a few studies have examined this mutual effect. The study conducted by Jocson among 81 Filipino low-income dyads indicated that parental depression predominantly affected rejection and harshness in their own parenting, without significantly influencing their partner’s harshness or rejection parenting (Jocson, 2021). Similarly, one study using a small sample of US couples with substance use disorder found that depressed parents were more likely to abuse their children, but did not affect partner abuse and over-reactivity parenting (Kelley et al., 2015).

The term “parenting” is defined as the relatively stable behavioral style and tendency exhibited by parents in the process of raising their children, which encompasses attitudes, beliefs, and behaviors towards their children (Parker, 1990). Previous studies have demonstrated that negative parenting aspects such as harshness, hostility, and intrusiveness affect children’s depression, internet addiction, and poor peer relationships (Chen et al., 2016; N. Jiang et al., 2021; L. Wang et al., 2022), while positive parenting promotes children’s mental health (Q. Jiang et al., 2024). Depression is theoretically defined as a psychological syndrome characterized by persistent low mood; loss of interest or pleasure; and a range of associated emotional, cognitive, physical, and behavioral symptoms. A large amount of evidence has demonstrated that parental depression exerts a significant impact on parenting behaviors (Cheung & Theule, 2019; Goodman et al., 2020). For example, depressive parents exhibited more conflict and ascribed negative interpretations to their children’s behaviors (Brenning et al., 2012). They were also more likely to experience negative emotions during parent–child interactions, diminish their perceptions of parenting efficacy, and resort to punishing their children (Wilson & Durbin, 2010). However, most previous studies have been conducted at the intraindividual level, i.e., the effect of maternal or paternal depression on their own parenting; thus the mutual influence of parental depression on parenting has rarely been investigated. In this study, parental depression was operationally defined as scores on the Center for Epidemiologic Studies Depression Scale (CES-D), reflecting the frequency of depressive symptoms (Radloff, 1977). Mutual influence refers specifically to the actor and partner effects within the Actor–Partner Interdependence Model (APIM), capturing the interdependence within the dyad where one’s characteristics can affect both one’s own and one’s partner’s outcomes.

To date, current researches have demonstrated the actor effect of parental depression on parenting, but the partner effect needs more studies to be revealed. Moreover, previous studies have ignored the potential role of parents’ gender. The “father vulnerability hypothesis” suggests that the father’s parenting is more susceptible to their partners’ depression than the mother’s parenting, given the secondary role of the father in parenting (Davies et al., 2009; Pedro et al., 2012). However, the situation may change with the increasing importance of fathering during adolescence. For example, one systematic review of the literature found that the father’s involvement increased during adolescence and had a positive effect on young development (X. Wang & Chi, 2025). Lastly, one limitation of existing research is that it mainly focuses on Western populations. The interdependence dynamic within families may differ across cultures. In Chinese families, although mothers are more involved in parenting during infant and childhood, fathers are equally involved in parenting adolescents (Li, 2021; Xu, 2021). Given the greater interdependence of caregivers in Chinese families, one caregiver’s depression may be related to both their own and their partner’s coparenting relationship quality, which can then affect both caregiver’s parenting behaviors (Chen et al., 2024).

This study aimed to investigate the mutual influence of parental depression and parenting (warmth–reasoning and harshness–hostility) among Chinese families. We used a large sample of families with twins from China, with parent and child reports. In two subsamples, the actor and partner effects of parental depression on warmth–reasoning and harshness–hostility were examined. We used two subsamples to cross-validate the findings. Children’s age and gender were controlled as covariates. Specifically, we planned to examine the influence of a parent’s depression on their own parenting (the actor effect) as well as on their spouse’s parenting (the partner effect). Three hypotheses were proposed in this study.

**Hypothesis 1.**  *The actor effect is significant. A parent’s depression positively predicts their own harshness–hostility and negatively predicts their own warmth–reasoning parenting.*

**Hypothesis 2.**  *The partner effect is significant. Maternal depression can affect the father’s parenting, and paternal depression can affect the mother’s parenting.*

**Hypothesis 3.**  *The partner effect of maternal depression is stronger than that of paternal depression.*

## 2. Method

### 2.1. Participants and Procedure

This study was based on data from the Beijing Twin Study (BeTwiSt), containing relevant information on teenagers and parents from 18 districts and counties in Beijing, China. BeTwiSt recruited twins and their parents from the urban and rural areas of Beijing. This study adopted a cross-sectional research design. Data collection was organized during after-school sessions in classroom settings. After explaining the research objectives and procedures, written informed consent was obtained from all participating parents. Participants with more than 80% missing data in the questionnaires were excluded. Trained research staff distributed the questionnaires and guided the adolescents to complete them independently. Parent questionnaires were completed at home. The sample for the current study comprised mother and father dyads (N = 1387), and two twin samples (N = 2774) (Chen et al., 2013). Multiple imputation (MI) was used to handle missing values, which utilized existing data information to fill in missing values and had high robustness (Woods et al., 2024). To avoid the impact of birth order, each child in one family was randomly selected to constitute a subsample. The age of the adolescents ranged from 8 to 21 years (mean = 13.61, SD = 2.72) and the sample comprised 52.3% females and 47.7% males. The study procedures and assessments were approved by the Ethics Committee of Institute of Psychology of CAS (Chinese Academy of Science), with IRB approval number IRB#H19049.

### 2.2. Measures

#### 2.2.1. Parenting Behaviors

The scale was adapted from the Iowa Youth and Families Project (Brody et al., 2001), which consisted of two subscales for paternal and maternal parenting styles, each comprising 32 items. The scale was organized into two dimensions: warmth–reasoning (13 items) and harshness–hostility (9 items). Warmth was defined as the parents’ support, understanding, appreciation, and concern for their children. Example items include “How often does your mother talk to you about things happening in life?” Reasoning refers to seeking children’s opinions and providing explanations. Example items include “How often does your mother discipline you by reasoning, explaining, or talking to you?” Harshness involves spanking the child, hitting them with a belt, or telling them to “get out”. Example items include “When you do something wrong, how often does your mother spank you?” Hostility encompasses criticizing, shouting, slapping, or swearing at children. Example items include “How often does your mother shout or yell at you because she is mad at you?” 

Adolescents and their parents rated parenting behaviors on a 5-point scale ranging from 1 (never) to 5 (always). A high reliability and validity of this scale was demonstrated in previous studies (Chen et al., 2016). In the present study, the Cronbach’s alpha coefficients for all dimensions were greater than 0.85, indicating a high degree of consistency, stability, and reliability.

#### 2.2.2. Depression Scale (CESD)

Parental depressive symptoms were measured using the 20-item Center for Epidemiologic Studies Depression Scale (CES-D; Radloff, 1977). Each item is rated on a 4-point scale (0–3) based on the frequency of symptoms experienced in the past week, with anchors ranging from 0 (“rarely or none of the time”) to 3 (“most or all of the time”). Four sample items are “I was bothered by things that don’t usually bother me”; “I felt depressed”; “I felt hopeful about the future”; and “I felt sad.” The Chinese version of the CES-D has been validated and shows good psychometric properties in Chinese adult populations. The higher the total score, the higher the parental depression level. In this study, the Cronbach’s alpha coefficient for paternal depression was 0.83, and the Cronbach’s alpha coefficient for maternal depression was 0.85, which could effectively measure parental depression levels.

### 2.3. Data Analysis

Data analyses were conducted using SPSS 22.0 for descriptive statistics and bivariate correlations, and Amos 22.0 for structural equation modeling. We constructed Actor–Partner Interdependence Models (APIMs) to estimate actor and partner effects between parental depression and parenting behaviors. Separate models were run for parent-reported and child-reported warmth–reasoning and harshness–hostility. Child age and gender were included as covariates. Model fit was assessed using chi-square, RMSEA, CFI, and NFI. The distinguishability test was conducted by comparison between saturated model and equality model. To quantify the relative strength of effects and determine the dyadic pattern, the *k* parameter (the ratio of partner effect to actor effect) was calculated within the APIM framework. A couple pattern is indicated when *k* equals or is close to 1, and a mixed pattern is found when *k* equals or is close to 0.5 (Kenny & Ledermann, 2010).

### 2.4. Common Method Deviation

To avoid the occurrence of common method bias effects, Harman’s single factor test was used (Zhou & Long, 2004). In two samples, the first common factor explained less than 40% of the total variance. Therefore, there was no significant common variance bias between the two samples and subsequent data analysis could be performed.

## 3. Results

### 3.1. Descriptive Statistics and Correlation Analysis

The distributions of the scores of study variables was approximately normal. The means, standard deviations, and correlation coefficients between parental depression and parenting behaviors are shown in Table 1. In two samples, depression in the father or mother was significantly negatively correlated with their own and their partner’s warmth–reasoning, and significantly positively correlated with their own and their partner’s harshness–hostility. The magnitudes of correlation are relatively larger in the data within reporters than across reporters (i.e., child vs. parent).

### 3.2. Dyadic Patterns Between Depression and Parenting

**Figure 1:**
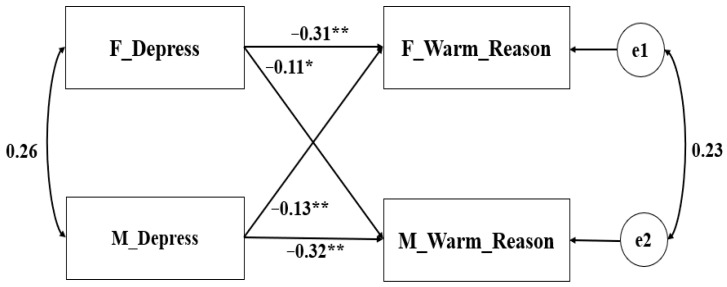
APIM of depression and warmth–reasoning reported by parents (Sample 1). ** *p* < 0.001, * *p* < 0.05.

**Figure 2:**
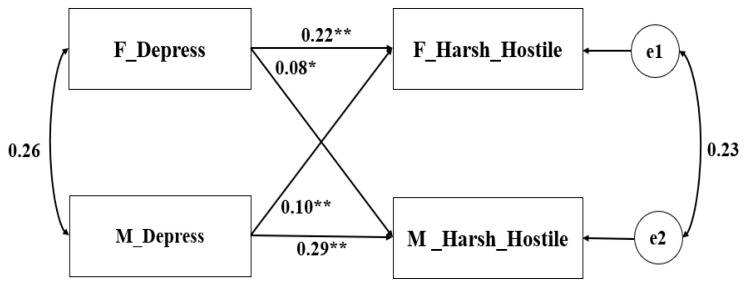
APIM of depression and harshness–hostility reported by parents (Sample 1). ** *p* < 0.001, * *p* < 0.05.

**Figure 3:**
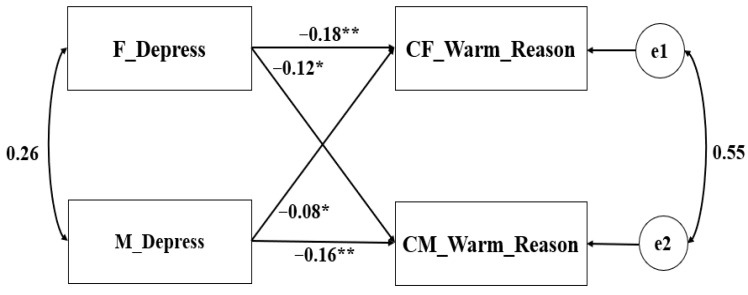
APIM of depression and warmth–reasoning reported by children (Sample 1). ** *p* < 0.001, * *p* < 0.05.

**Figure 4:**
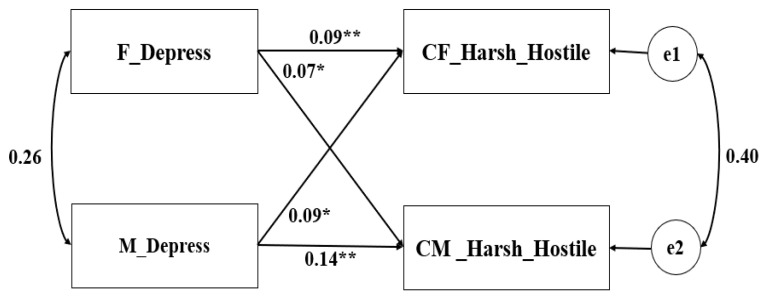
APIM of depression and harshness–hostility reported by children (Sample 1). ** *p* < 0.001, * *p* < 0.05.

Theoretically, fathers and mothers could be distinguished based on gender. Consequently, the pairwise distinguishable relationships were tested in the APIM (Liu & Wu, 2017). Additionally, the gender and age of the child were taken into account as covariates. Parental depression served as the predictor variable, and the parenting behaviors (warmth–reasoning and harsh–hostility) were the outcome variables in the constructed model. The saturated models revealed that both the actor and partner effects were all significant (see Table 2). The non-standardized coefficient results are depicted in Figure 1, Figure 2, Figure 3 and Figure 4.

Regarding parental depression and parent-reported parenting, the model fitting was deemed satisfactory across various indicators: χ^2^/df = 1.855 < 3, RMSEA = 0.025 < 0.1, CFI = 0.993 > 0.9, NFI = 0.986 > 0.9. The magnitudes of the actor effects (i.e., effect of a parent’s depression on their own parenting) look larger than those of the partner effects (i.e., effect of a parent’s depression on their partner’s parenting). We then statistically tested whether the actor and partner effects are equal with APIM including the ghost variables. For the effects of paternal depression, the *k*(f) = 0.404 (95% CI: 0.200~0.702), while for the effects of maternal depression, *k*(m) = 0.332 (95% CI: 0.128~0.611). The confidence interval encompassed 0.5, indicating that the paired pattern of parents was a mixed mode, which indicated that the actor effect of depression on warmth–reasoning (parent-reported) was greater than the partner effect.

Following the same steps, the model was constructed with parental depression as the independent variable and harshness–hostility (parent report) as the dependent variable (as illustrated in Figure 2). The model fitted well and the actor and partner effects were all significant. For the effects of paternal depression, *k*(f) = 0.442 (95% CI: 0.169~0.852), while for maternal depression, *k*(m) = 0.297 (95% CI: 0.092~0.556). The confidence intervals of the *k* parameter encompassed 0.5, indicating that the paired pattern of parents was also a mixed mode.

We further verified our results using child-reported parenting. The results are presented in Figure 3 and Figure 4. Overall, the fitting indicators of each model reached the ideal level. To test the paired patterns between parental depression and parenting behaviors (child-reported), an APIM model with ghost variables was constructed. The results showed that parental depression had a significant impact on both a parent’s own and their partner’s warmth–reasoning and harshness–hostility. However, the relative strengths of the actor effect and partner effect seem more similar, as the confidence intervals of the *k* parameter encompassed both 0.5 and 1. These findings should be interpreted with caution, since both the father’s and mother’s parenting behaviors were reported by the same child.

To cross-validate the findings, we conducted the same statistical analyses with the subsample (sample 2) with twin siblings from each same family. Thus, sample 2 is very similar to sample 1 regarding genetic makeup, family background, age range, and parental characteristics. The results from sample 2 are provided in the Supplementary Materials. Similar to the main findings, the findings from sample 2 also indicated that parental depression significantly impacted a parent’s own and their partners’ parenting. Overall, these findings supported research hypotheses 1 and 2. However, we found that the partner effect of maternal depression and paternal depression seemed similar; thus, research hypothesis 3 was not supported.

## 4. Discussion

This study examined the dyadic relations between depression and parenting in a large sample of Chinese families with adolescent twins. Across both parent- and child- reported data, we found that depression in a parent not only affected their own warmth–reasoning and harshness–hostility (actor effect), but also affect their partner’s warmth–reasoning and harshness–hostility (partner effect).

The hypothesis regarding the actor effect of depression in a parent on their own parenting was supported. We found that depression in both the mother and father, respectively, significantly correlated with more harshness–hostility and less warmth–reasoning. These findings are consistent with previous studies conducted at the intraindividual level (Cheung & Theule, 2019; Goodman et al., 2020; Frost et al., 2025). Depressed parents tend to exhibit more conflict and ascribe negative interpretations to their children’s behaviors (Brenning et al., 2012), which may evoke more negative parenting behavior. Parents with a higher level of depressive symptoms are also more likely to experience negative emotions during parent–child interactions, diminish their perceptions of parenting efficacy, and thus lessen their positive parenting behavior (Wilson & Durbin, 2010).

Previous studies based on small samples from the United States and the Philippines found that the partner effect of depression on parenting was not significant (Jocson, 2021; Rubin & Kelly, 2015). However, the current study, based on one large sample of Chinese families and multiple reports, found a significant partner effect of both maternal depression and paternal depression, thus supporting our second hypothesis. In Chinese collectivist culture, both the mother and father are more involved in parenting their offspring, especially during the adolescent period (Li, 2021). The greater interdependence of coparenting may amplify the mutual influence between Chinese family members. Thus, one caregiver’s depressive symptoms may elicit a poor-quality coparenting relationship, which will then make another caregiver more likely to resort to harsh discipline rather than warmth (Chen et al., 2024; Luo et al., 2025).

Regarding the relative strength of the actor effect versus the partner effect, the findings vary according to the informants of parenting. Specifically, with the association between parent-reported depression and parent-reported parenting, the actor effects are larger than the partner effects. This finding indicates that parental depression influences their own parenting more than their spouse’s parenting. On the other hand, with the association between parent-reported depression and child-reported parenting, the partner effect is still less than the actor effect, but the difference becomes smaller. This may be due to the same child reporting both their father’s and mother’s parenting. Together, these findings support the family systems theory, which posits that family members are interdependent, and one parent’s intrapersonal depression can not only affect their own interaction with the child, but also exert an influence on their partner’s interaction with the child (Cox & Paley, 2003).

Furthermore, we found no significant difference in the impact of paternal versus maternal depression on a couple’s parenting, which did not support the father vulnerability hypothesis. One possible reason for this may be that Chinese fathers were more involved in their children’s lives than in previous generations and had a clearer father role, especially for adolescents (Karnilowicz et al., 2019). Additionally, the results of a qualitative study indicated that Chinese fathers did not appear to be concerned about asserting parental authority or embodying the emotionally stoic masculine norm. In contrast, they willingly demonstrated their parental love and affection, emphasizing child-centeredness and the socioemotional well-being of their children (Li, 2021).

The current study has the strengths of a large sample size and multiple reporters, but there are still several limitations. First, all the results of the present study were drawn from a sample of families with adolescent twins; thus, it is unclear if the results could be generalized to other ethnic groups. Second, all study measures relied on subjective reports, so the results may have subjective bias. Third, the study design is cross-sectional; therefore, potential reciprocal relations between parental depression and parent–child interactions need to be considered in future longitudinal research.

## 5. Practical Implications and Suggestions for Future Research

The findings have practical significance for academic research and family support. This is the first study based on Chinese twins to find that depression in a parent can significantly affect their own and their partner’s warmth–reasoning and harshness–hostility, thereby providing new perspectives and empirical evidence for research on parenting. Additionally, the results indicated that, within the context of Chinese culture, depression in a parent exerted a more pronounced influence on their partner’s parenting compared to Western samples. This suggests the need for the development of targeted parenting interventions for Chinese families. In improving parenting quality, reducing parental depression could be adopted as a pivotal point of intervention. By considering the father, mother, and child as an integrated family system, a variety of psychological regulation strategies could be employed to alleviate parental depression. Social service organizations could provide specialized and personalized mental health services and emotional support to parents experiencing depression, thereby enhancing their mental well-being and optimizing the family’s parenting environment.

Future empirical studies are needed to systematically explore the mediating and moderating variables between depression and parenting, such as child characteristics, marriage quality, couple interaction, parental conflict, emotion regulation, parenting attitudes, and so on. Second, the role of extended family members, such as relatives, grandparents and so on, also needs to be taken into consideration in future studies. The current study adopts a cross-sectional design, and it is imperative to conduct future research utilizing longitudinal data to ascertain the causality between depression and parenting.

## 6. Conclusions

This study employed the Actor–Partner Interdependence Model (APIM) to investigate the mutual influence between parental depression and parenting behaviors (warmth–reasoning and harshness–hostility) in a large sample of 1387 Chinese adolescent twin families, with cross-validation using a subsample of twin siblings. We found that depression in a mother or father can influence not only their own parenting behaviors, but also their spouse’s parenting. These findings not only enrich theoretical frameworks (e.g., Family System Theory) in cross-cultural contexts but also offer empirical guidance for family-based intervention—emphasizing that addressing parental depression should target both spouses as an integrated system to optimize parenting quality and support adolescent development.

## Figures and Tables

**Table 1:** Descriptive statistics and correlation matrix of parental depression and parenting.

Sample	Dimension	M ± SD	CF-W	CF-H	CM-W	CM-H	F-W	F-H	M-W	M-H
1	Paternal Depression	−0.619 ± 0.856	−0.188 **	0.119 **	−0.156 **	0.110 **	−0.313 **	0.246 **	−0.199 **	0.173 **
	Maternal Depression	−0.423 ± 0.886	−0.127 **	0.110 **	−0.177 **	0.151 **	−0.207 **	0.167 **	−0.325 **	0.300 **
2	Paternal Depression	−0.619 ± 0.856	−0.184 **	0.150 **	−0.147 **	0.134 **	−0.262 **	0.213 **	−0.182 **	0.127 **
	Maternal Depression	−0.423 ± 0.886	−0.120 **	0.107 **	−0.178 **	0.154 **	−0.200 **	0.164 **	−0.302 **	0.239 **

Note: ** *p* < 0.001, CF-W: fathers’ warmth–reasoning reported by children; CF-H: fathers’ harshness–hostility reported by children; CM-W: mothers’ warmth–reasoning reported by children; CM-H: mothers’ harshness–hostility reported by children; F-W: warmth–reasoning reported by father; F-H: harshness–hostility reported by father; M-W: warmth–reasoning reported by mother; M-H: harshness–hostility reported by mother.

**Table 2:** Summary of APIM for depression and parenting (Sample 1).

Variable	a1	a2	p12	p21	Saturated Model				Equality Model		Model with Ghost Variables				p12 = p21		Mode
					χ^2^/df	RMSEA	CFI	NFI	χ^2^/df	*p*	k1 (95%CI)	k2 (95%CI)	χ^2^/df	*p*	χ^2^/df	*p*	
P-W	−0.31 ** (−0.27 **)	−0.32 ** (−0.29 **)	−0.13 ** (−0.12 **)	−0.11 ** (−0.09 *)	1.855	0.025	0.993	0.986	0.097	0.908	0.404 [0.200, 0.702]	0.332 [0.128, 0.611]	0.011	1.000	0.096	0.962	Mixed Mode
P-H	0.22 ** (0.21 **)	0.29 ** (0.28 **)	0.10 ** (0.10 **)	0.08 * (0.08 *)	1.855	0.025	0.993	0.986	1.499	0.223	0.442 [0.169, 0.852]	0.297 [0.092, 0.556]	0.051	0.995	0.133	0.940	Mixed Mode
C-W	−0.18 ** (−0.18 **)	−0.16 ** (−0.14 **)	−0.08 * (−0.07 *)	−0.12 ** (−0.10 *)	1.855	0.025	0.995	0.990	0.311	0.733	0.432 [0.076, 1.045]	0.748 [0.260, 1.559]	0.258	0.905	0.952	0.414	Mixed Mode
C-H	0.09 * (0.09 *)	0.14 ** (0.13 **)	0.09 * (0.08 *)	0.07 * (0.06 *)	1.855	0.025	0.993	0.986	0.681	0.506	0.904 [0.185, 1.791]	0.500 [0.043, 1.374]	0.103	0.981	0.089	0.966	Mixed Mode

Note: ** *p* < 0.001, * *p* < 0.05. P-W: warmth–reasoning reported by parents; P-H: harshness–hostility reported by parents; C-W: warmth–reasoning reported by children; C-H: harshness–hostility reported by children. a1: the actor effect of father’s depression on father’s parenting; a2: the actor effect of mother’s depression on mother’s parenting; p12: the partner effect of mother’s depression on father’s parenting; p21: the partner effect of father’s depression on mother’s parenting. Mixed Mode: Both actor and partner effects are significant, and the actor effect is greater than the partner effect. Saturated model: Free estimation in the model; Equality model: limit a1 = a2, p12 = p21 in the model. Model with ghost variables: The ratio *k* of partner effect to actor effect is used to determine the dyadic pattern.

## Data Availability

The datasets generated and analyzed during the current study are not publicly available but are available from the corresponding author on reasonable request.

## Supplementary Materials

The following supporting information can be downloaded at: https://www.mdpi.com/article/10.3390/bs16010103/s1, Figure S1. APIM of depression and warmth-reasoning reported by parents. Figure S2. APIM of depression and harshness-hostility reported by parents. Figure S3. APIM of depression and warmth-reasoning reported by children. Figure S4. APIM of depression and harshness-hostility reported by children. Table S1. Summary of APIM for depression and parenting (Sample 2).
