# Supplementary material for: Mutual Influence of Parental Depression and Parenting: An Actor–Partner Interdependence Analysis Based on Chinese Families with Adolescent Twins
Source: Behav Sci (Basel). 2026 Jan 12;16(1):103. doi: 10.3390/bs16010103 (PMC12838220; doi:10.3390/bs16010103)
Supplement: Supplementary file 1 [file behavsci-16-00103-s001.zip › behavsci-4013459-supplementary.pdf]

Supplementary Materials (Results of the subsample 2)

Depression was served as the predictor variables, and the warmth-reasoning (parent report) were the outcome variable in the constructed model. The model fitting was deemed satisfactory across various indicators: $\chi^2/df = 1.855 < 3$, RMSEA = 0.025 < 0.1, CFI = 0.993 > 0.9, NFI = 0.985 > 0.9. The non-standardized coefficient results were depicted in Figure S1. The saturated model revealed that both the actor and partner effects were significant.

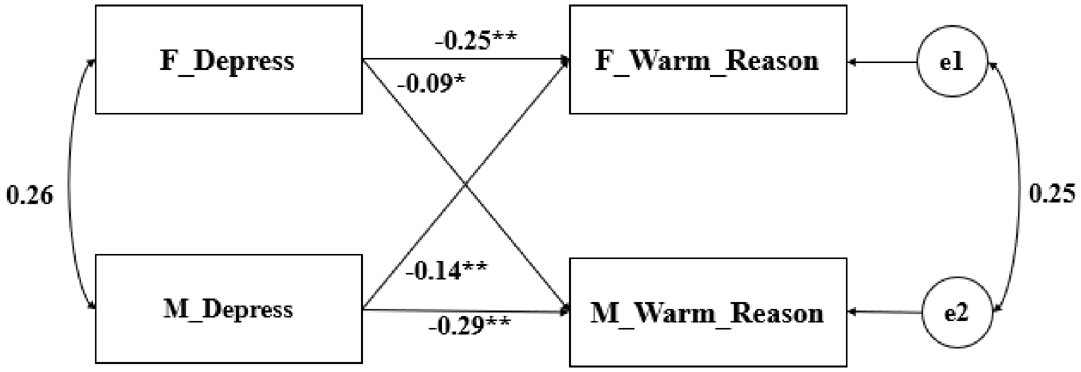

Figure S1. APIM of depression and warmth-reasoning reported by parents

We then examined whether the actor and partner effect are equal through APIM that included ghost variables. The results showed that father k1=0.557 (95% CI: 0.282~0.975), mother k2=0.311 (95% CI: 0.106~0.594). To verify the paired pattern of parents, k was constrained special values within the confidence interval (father k = 0.5, mother k = 0.5). The X^2 variation of the model was not significant, $\chi^2/df=0.489$, $p=0.744>0.05$. Therefore, the paired patterns were mainly belonged to mixed mode, which indicated that the actor effect of depression on warmth-reasoning was greater than the partner effect.

Follow the same steps, the model was constructed with depression as the independent variable and harshness-hostility (parent report) as the dependent variable (as illustrated in Figure S2). The model fitted well and the actor and partner effect were significant. When constraining the parent's actor and partner effects in the model to be

equal, we found χ^2/df=0.951, p=0.386>0.2, which suggested that the data could be treated as distinguishable. The model with ghost variables showed that father k1=0.585 (95% CI: 0.223~1.140), mother k2=0.231 (95% CI: 0.008~ 0.551). The confidence interval included 0.5, indicating that the paired pattern of parents was a mixed pattern.

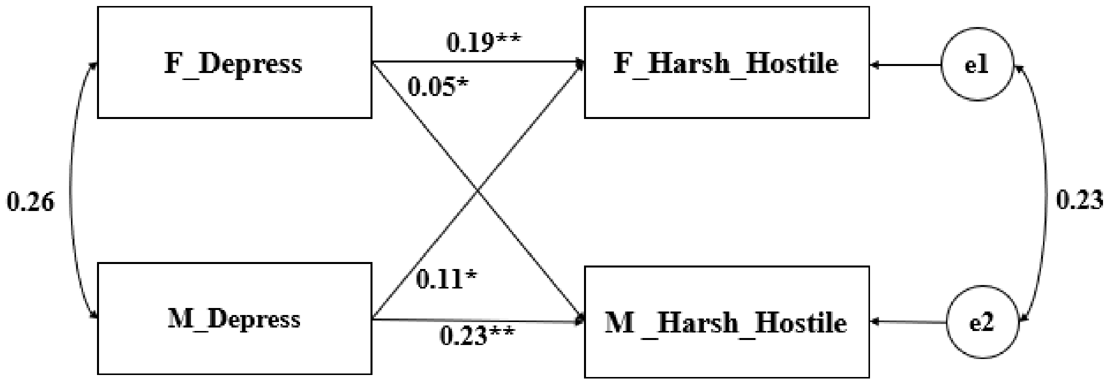

Figure S2. APIM of depression and harshness-hostility reported by parents

The results reported by children were presented in Figure S3 and Figure S4 and the specific data analysis results were presented in Table S1. Numbers were unstandardized estimates shown in the table and the numbers in parentheses were standardized estimates. Overall, the fitting indicators of each model reached the ideal level. The warmth-reasoning and harshness-hostility reported by children could be considered as indistinguishable data for further analysis (p>0.2). To test the paired patterns of parenting, an APIM model with ghost variables was constructed. The results showed that depression had a significant impact on both self and partner's warmth-reasoning and harshness-hostility. The paired patterns were mainly belonged to mixed mode, which indicated that the actor effect of depression on parenting was greater than the partner effect. Additionally, there was no significant difference between p_{12} and p_{21} (p>0.05). That was to say, there was no significant difference in the partner effect between fathers and mothers.

Table S1. Summary of APIM for depression and parenting (Sample 2)

Variable	a1	a2	p12	p21	Saturated model				Equality model		Model with ghost variables				p12=p21		Mode
					X^2/df	RMSEA	CFI	NFI	X^2/df	p	k1 (95%CI)	k2 (95%CI)	X^2/df	p	X^2/df	p	
P- W	**-0.25**** (-0.22**)	**-0.29**** (-0.27**)	**-0.14**** (-0.13**)	**-0.09*** (-0.08*)	**1.855**	**0. 025**	**0.993**	**0.985**	**0.654**	**0.520**	**0.557** [0.282, 0.975]	**0.311** [0.106, 0.594]	**0.489**	**0.744**	**1.339**	**0.260**	**Mixed Mode**
P- H	**0.19**** (0.18**)	**0.23**** (0.23**)	**0.11**** (0.11**)	**0.05*** (0.05*)	**1.855**	**0. 025**	**0.993**	**0.985**	**0.951**	**0.386**	**0.585** [0.223, 1.140]	**0.231** [0.008, 0.551]	**0.017**	**0.999**	**1.172**	**0.319**	**Mixed Mode**
C-W	**-0.18**** (-0.16**)	**-0.16**** (-0.15**)	**-0.07*** (-0.06*)	**-0.10**** (-0.09**)	**1.855**	**0. 025**	**0.995**	**0.989**	**0.213**	**0.809**	**0.391** [0.043, 0.927]	**0.647** [0.218, 1.420]	**0.226**	**0.924**	**0.398**	**0.754**	**Mixed Mode**
C-H	**0.13**** (0.12**)	**0.13**** (0.12**)	**0.07*** (0.07*)	**0.10*** (0.09*)	**1.855**	**0. 025**	**0.994**	**0.986**	**0.313**	**0.732**	**0.518** [0.058, 1.465]	**0.733** [0.213, 1.831]	**0.125**	**0.974**	**0.356**	**0.785**	**Mixed Mode**

PS: **p<0.001, *p<0.05. **P-W**: warmth-reasoning reported by parents; **P-H:** harshness-hostility reported by parents; **C-W:** warmth-reasoning reported by children; **C-H:** harshness-hostility reported by children. **a1**: the actor effect of father's depression on father's parenting; **a2:** the actor effect of mother's depression on mother's parenting; **p12:** the partner effect of mother's depression on father's parenting; **p21**: the partner effect of father's depression on mother's parenting. **Mixed Mode**: Both actor and partner effects are significant, and the actor effect is greater than the partner effect. **Saturated model**: Free estimation in the model; **Equality model:** limit a1=a2, p12=p21 in the model; **Model with ghost variables:** The ratio k of partner effect to actor effect is used to determine the dyadic pattern. There is no significant difference between p12 and p21.

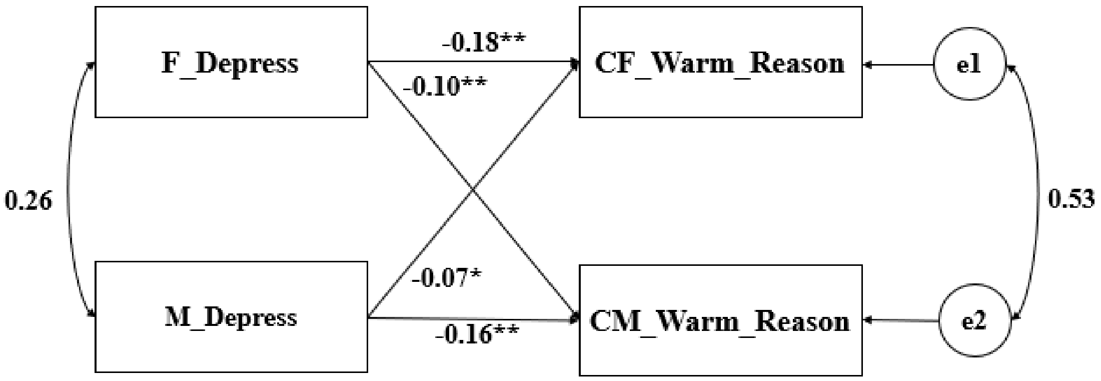

Figure S3. APIM of depression and warmth-reasoning reported by children

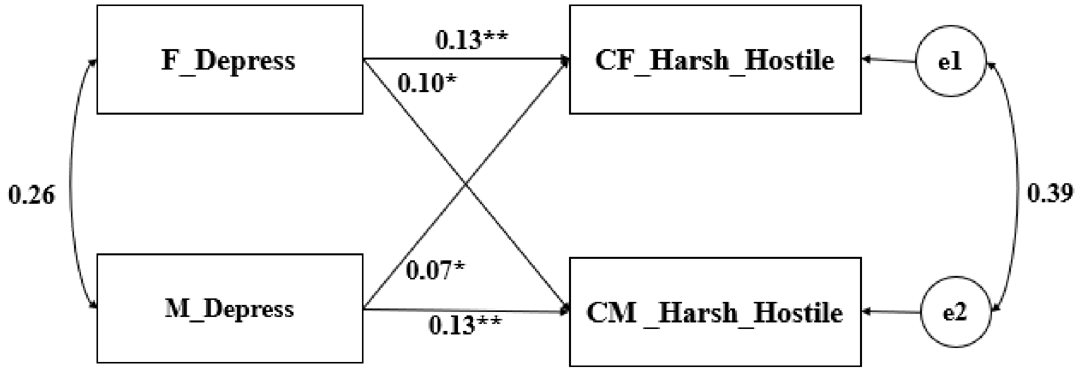

Figure S4. APIM of depression and harshness-hostility reported by children
